# The response to individualized treatment after a standardized treatment protocol among neck pain sufferers: a secondary analysis of a randomized controlled trial

**DOI:** 10.1186/s12998-025-00579-y

**Published:** 2025-04-11

**Authors:** Anders Galaasen Bakken, Andreas Eklund, Anna Oksanen, Iben Axén

**Affiliations:** 1 Unit of Intervention and Implementation Research for Worker Health, Department of Environmental Medicine, Karolinska Institutet, Nobels Väg 13, 171 77 Stockholm, Sweden; 2 Division of Biostatistics, Karolinska Institutet, Nobels Väg 13, 171 77 Stockholm, Sweden

**Keywords:** NRS-11, Persistent neck pain, Responders, SMT, Spinal manipulative therapy, Neck disability

## Abstract

**Background:**

Manual therapy and exercise are recommended for patients with neck pain. In a recent randomized controlled trial, home stretching exercises with or without manual therapy were offered to subjects with persistent or recurrent neck pain. No difference in pain or disability between the treatment groups were found after the two-week intervention period. We aimed to investigate whether these patients had a better outcome after individual tailoring of the treatment content two months after the initial structured intervention period.

**Methods:**

This manuscript is a secondary analysis of a previous clinical trial where 131 patients with persistent or recurrent neck pain received treatments over two weeks (the intervention period). Pain and disability were assessed for two months following the intervention period. During this period, the treating therapists could recommend further individualized tailored treatment, including any treatment modality, regardless of the intervention group and whether the participants responded to the intervention (responders) or not (non-responders). Responders from the intervention period were defined as reporting a minimal clinical improvement on the numeric rating scale (NRS-11) at a 20-percentage points improvement (2 increments), regardless of group belonging in the original trial. All other participants were considered non-responders. We also evaluated the number of treatments, differences in disability, quality and affective component of pain, and quality of life during the individualized care period.

**Results:**

For responders to a randomized trial of manual therapy and stretching exercises, a significant worsening in pain was associated with an increasing number of treatments during a two-month individualized care period. Among non-responders to the initial intervention period, improvement in neck pain disability was observed with individually tailored treatments.

**Conclusions:**

For responders to a randomized trial of manual therapy and stretching, worsening pain in the individualized care period was associated with increasing numbers of individually tailored treatments. Among non-responders to the initial intervention period, improvement in neck pain disability was observed with individually tailored treatments.

*Trial registration*: The trial was registered at ClinicalTrials.gov, registration number NCT03576846, on 23rd of June 2018.

**Supplementary Information:**

The online version contains supplementary material available at 10.1186/s12998-025-00579-y.

## Introduction

A recent systematic review of the European treatment guidelines for neck pain (NP) ranks topical medications, manual therapy + other treatments, exercise programs, and exercise therapy + other treatments as the highest recommended treatment modalities (moderate evidence) [1].

Manual therapy and exercise have been reported to be beneficial for persistent NP compared to no treatment [2, 3], but a combination of the two show conflicting results [4–6]. No favorable effect has been observed when comparing manual therapy and exercises vs. exercises alone [2, 3] or vs. massage [7] after a four-week follow-up. There are conflicting reports on the long-lasting effect of therapeutic interventions for NP [8, 9], and further research on the long-term effects is needed [5]. In a recent randomized controlled trial, no additional effect of Spinal Manipulative Therapy (SMT) and home stretching exercises over home stretching exercises alone was reported after a two-week intervention period [10–13].

It has been debated for some time that the results of systematically applied therapies may be inappropriate to test their effectiveness [12] due to the heterogeneity of pain populations. Arguably, people will respond differently to interventions. In the real world, clinicians will assess each patient’s needs and preferences and not offer the same treatment modalities and number of treatments to all patients with the same diagnosis. Factors that affect the outcome of an intervention could be age, pain intensity, co-morbidity, and distribution of pain [14]. Before deciding on treatment, the clinician considers patient expectations, psychosocial risk factors, and treatment preferences. However, the clinical implications of individually tailoring the content of manual intervention (such as SMT) have not been investigated.

The effect of individual exercise plans on NP is inconclusive due to a limited number of studies. Nevertheless, a systematic review found no short-term effect over general exercises [15, 16] but some effect over general exercises in the long term [16].

We recently conducted a trial where participants with persistent or recurrent NP were given home stretching exercises with or without manual therapy during a 2-week intervention period. We found no difference in pain or disability between the treatment groups at two weeks [12]. After the intervention period, the clinicians could tailor treatment modalities and management strategies to each patient if deemed appropriate. The clinician would base this decision on their clinical judgment, irrespective of intervention during the intervention period or effect observed. The participants were also free to decline or accept any offer of further individually tailored treatment.

The primary aim of this study was to investigate if responders and non-responders to the initial intervention would respond to an individually tailored treatment plan by reporting improvement in pain intensity, disability, quality and affective component of pain, and quality of life. Based on previous research [17], we expected the improvements in pain intensity reported immediately after the intervention period to remain in the two-month individualized care period. Based on current guidelines, the group not receiving manual therapy during the intervention period could benefit from including manual therapy after the intervention period [18–21].

In addition, the number of treatments associated with changes in pain intensity during the individualized care period was investigated. This information is essential as it can guide clinicians in designing continuing treatment plans for non-responders after an initial intervention.

The two-month outcomes of the original RCT with respect to changes in pain intensity, disability, quality and affective component of pain, and quality of life will also be reported, as this information has not been included in previous publications.

## Methods

This is a secondary analysis of a randomized controlled trial (RCT) where participants with persistent NP were booked for four appointments with a chiropractor over two weeks. Recruitment began in January 2019, with all follow-up questionnaires answered by June 2020.

The intervention group received home stretching exercises and SMT, while the control group only received home stretching exercises. All patients received the same number of examinations and appointments with the chiropractor. As reported elsewhere, the compliance was good; all participants completed their stretching exercises at least 10 out of 14 days [12]. After the intervention period, all participants were recommended further individually tailored treatments if deemed appropriate by their treating chiropractor. This could for example have been recommended if the participant was not improving or if the improvement did not meet the treatment goals. Patients were asked to rate pain intensity (numeric rating scale, NRS-11), quality and affective component of pain (McGill questionnaire), neck disability (Neck disability index), and generic health status (EQ-5D) every two weeks over a two-month individualized care period after the initial intervention period ended.

The study was a multicentre RCT utilizing primary care rehabilitation clinics in Stockholm, Sweden. The clinics were all part of the regional health service, meaning that all included patients only paid part of the incurred fee, as usual when seeing a health care professional in this setting.

### Inclusion/exclusion criteria

Participants were included if they were over 18 years of age, had experienced NP for more than six months (self-reported), and had received no chiropractic treatment in the previous three months.

Participants were excluded if they had a medical condition that could affect the heart rate variability measurements [13], as this was an outcome in the trial, reported elsewhere [11], or present contraindications to manual therapy [22].

### Consent

All patients received information on the study both in writing and verbally. Signed consent was obtained from all participants before inclusion in the trial.

### Randomization

The patients were randomized using a 1:1 allocation ratio in randomly permuted different-sized blocks. An independent statistician prepared the randomization sequence off-site in sealed envelopes with instructions.

### Clinic visits

At the baseline visit, patients answered questionnaires regarding their pain (site, intensity, duration, STaRT Back prognosis, sick-leave, expectations for improvement), disability, and demographic information (age, sex, previous experience with chiropractic, type of work). A research assistant collected study data before patients were sent on their first visit with their chiropractor, who then unveiled which group the patient was allocated to.

### Blinding

All patients were blinded to the other treatment group. The statistician and research assistants were blinded to treatment allocation. As the clinicians provided treatment in both groups, blinding was not possible.

### Outcome measures

The outcomes of the RCT are reported elsewhere [11–13]. For this study, the following variables were used in the analysis:

### Pain

Pain was measured using an 11-point numeric rating scale (NRS-11) [23, 24] and a short-form McGill questionnaire [25, 26] to cover both pain intensity, quality and effective components of pain. All questionnaires are validated in Swedish [23–29].

### Disability

Disability was measured using the neck disability index (NDI) [30], a reliable and validated measure of NP disability [30].

### Health-related quality of life

The EQ-5D was used to measure health-related quality of life. The EQ-5D has been validated people with persistent pain, measuring secondary effects [31] and is reliable and valid in the Swedish language translation [28, 29].

All measurements were recorded weekly during the intervention period, and NRS-11 was measured daily using text messages. After the intervention period, all outcome questionnaires (Online Appendix 1) were emailed to respondents every two weeks for two months. The questionnaires were web-based.

### Treatment content and number of treatments

Treatment content was reported after the data collection ended. The participating chiropractors used their journal notes to report the number and dates of treatments during the individualized care period and the different treatment modalities utilized for each case.

### Statistical analysis

Categorical variables were reported as counts and percentages, and continuous variables with means and standard deviations.

Intention to treat analysis was used, but participants who dropped out (n = 5) before the two-month individualized care period could not be included in any of the analyses. During the intervention, patients were told to ignore questions unrelated to their pain experience in the McGill questionnaire. Hence, single questions not answered within the questionnaire were assumed to have a zero value. Pain intensity using NRS-11 was extracted from the McGill questionnaire. Missing values from the NDI and EQ-5D instruments were imputed using multiple imputations with fully conditional specifications and twenty imputation rounds [32].

*P*-values smaller than 0.05 were considered statistically significant.

The figures of responder/non-responder, sex, and intervention/control groups used predictions from a mixed model with person-specific random intercept and an interaction term for week and groups. The software used was Stata, version 15 (StataCorp. 2017. Stata Statistical Software: Release 15. College Station, TX: StataCorp LLC).

Groups were created based on the minimal clinical important difference (MCID) for the NRS-11 measure at the end of the intervention period. The threshold for the responder category (MCID) was set at 20 percentage points (≥ 2 increment change) [33, 34]; all other participants were considered non-responders. Only individuals who received treatments during the individualized care period were included.

The cohort was also stratified based on intervention, control, and sex.

A linear mixed effects model with a person-specific random intercept had the best fit, as reported in the previous article investigating short-term outcomes [12]. Depending on the model, the estimate of change with time (speed of change) or the interaction between time and group were the parameters of interest, as in the previous article [12]. An additional analysis, adjusted for age, sex, and baseline values, was also performed.

Using mixed linear regression models, all of the mentioned groups (responder/non-responder, intervention/control, male/female) were analyzed for the outcome pain intensity (NRS-11); in addition, responder/non-responder, intervention/control, were also analyzed for the outcomes quality and affective component of pain (McGill Questionnaire), disability (NDI), and generic health status (EQ-5D). A univariate linear regression model was also used to investigate the relationship between the number of treatments and changes in NRS-11. Treatment modalities were reported descriptively.

## Results

### Participants

The study group consisted of 126 participants, slightly more females than males, with a mean age of 56 years. Most experienced pain in other body areas and had suffered from NP for several years.

The baseline characteristics for the original intervention and control groups, as well as the comparative groups, can be found in Table 1.

**Table 1:** Baseline demographics for all comparative groups

	Intervention (66)	Control (60)	Women (70)	Men (56)	Responders (43)*	Non-responders (83)*
Year born, Mean (sd)	1964 (14.0)	1962 (13.8)	1964 (14.6)	1962 (13.0)	1965 (15.1)	1962 (13.2)
Female, n (%)	37 (56)	33 (55)			23 (53)	47 (57)
Pain duration						
1. Less than 6 months, n (%)	0 (0)	1 (2)	0 (0)	1 (2)	0 (0)	1 (1)
2. 6–12 months, n (%)	8 (12)	10 (17)	9 (13)	9 (16)	6 (14)	12 (15)
3. Several years, n (%)	57 (88)	47 (81)	59 (87)	45 (82)	36 (86)	68 (84)
STarT Back categories						
1. Low risk, n (%)	47 (80)	44 (79)	46 (73)	45 (87)	33 (83)	58 (77)
2. Medium risk, n (%)	7 (12)	11 (20)	13 (21)	5 (10)	5 (13)	13 (17)
3 High risk, n (%)	5 (8)	1 (2)	4 (6)	2 (4)	2 (5)	4 (5)
If seen a chiropractor before, how effective was it?						
1. Never seen a chiropractor before, n (%)	12 (18)	11 (18)	12 (17)	11 (20)	6 (14)	17 (21)
2. Good or excellent, n (%)	39 (60)	31 (52)	39 (56)	43 (77)	6 (14)	12 (15)
3. No difference, n (%)	14 (22)	17 (28)	17 (25)	10 (18)	23 (53)	29 (35)
4. Got worse, n (%)	0 (0)	1 (2)	1 (1)	0 (0)	8 (19)	23 (28)
Type of occupation						
1. No job, n (%)	19 (29)	20 (33)	22 (31)	17 (30)	13 (30)	26 (31)
2. Mostly hard labour, varied or standing, n (%)	17 (27)	14 (24)	16 (23)	15 (27)	13 (30)	18 (39)
3. Mostly sitting, n (%)	30 (45)	26 (43)	32 (46)	24 (43)	17 (40)	39 (47)
Arm pain, n (%)	42 (65)	32 (55)	45 (66)	29 (53)	26 (61)	48 (61)
Pain in the midback, n (%)	24 (39)	22 (39)	45 (65)	28 (56)	21 (51)	52 (67)
Pain in the low back, n (%)	25 (39)	24 (41)	45 (66)	28 (52)	22 (54)	51 (63)
Sick leave the previous year						
Do not work, n (%)	13 (20)	18 (30)	17 (24)	14 (25)	11 (26)	20 (24)
No, n (%)	47 (71)	36 (60)	42 (60)	41 (73)	30 (70)	53 (64)
Yes, between 1–7 days, n (%)	3 (5)	2 (3)	5 (7)	0 (0)	2 (5)	3 (4)
Yes, between 8–14 days, n (%)	3 (5)	0 (0)	2 (3)	1 (2)	0 (0)	3 (4)
Yes, more than 15 days, n (%)	0 (0)	4 (7)	4 (6)	0 (0)	0 (0)	4 (5)
Expect to improve (0–10), Mean (std)	6.0 (2.2)	5.7 (2.4)	5.9 (2.3)	5.8 (2.4)	6.3 (2.3)	5.7 (2.3)
Baseline NRS-11 Mean, (std)	4.7 (2.0)	4.2 (2.2)	4.8 (2.2)	4.0 (1.9)	5.5 (1.7)	3.9 (2.1)

*Responders: ≥ 2/10 NRS-11), Non-responders: ≤ 1/10 NRS-11

The response rate for each outcome for each time point is found in Online Appendix 2.

A total of 92/126 patients received at least one additional treatment during the individualized care period and were included in further analysis. Nearly all of these received some form of manual therapy. The number of participants receiving additional treatment is found in Table 2.

**Table 2:** Number of participants receiving additional treatment in the individualized care period stratified by intervention/control, responders/non-responders, and sex

	Number of patients receiving additional treatment	Mean number of treatments (SD)	Median number of treatments (IQR)
Non-responders	63	2.9 (1.5)	3 (2–4)
Responders	29	2.7 (1.4)	2 (2–3)
Intervention	44	2.6 (1.5)	2 (1–3)
Control	48	3.1 (1.5)	3 (2–4)
Males	38	2.6 (1.5)	2 (1–3)
Females	54	3.0 (1.5)	3 (2–4)
Total	92	2.8 (1.5)	3 (2–4)

The treatment modalities are described in Table 3.

**Table 3:** Overview of treatment modalities the chiropractors utilized in the treatments following the intervention period

Treatment modality	n of participants
One or more joint-based treatment technique (Gonstead^a^, Diversified^b^, activator^c^, traction^d^, blocking^e^, mobilization^f^)	85
Rehabilitating exercises (Improve physical function by patients own effort)	29
Trigger points treatment (Pressure on tension spots in a muscle)	45
Acupuncture (the insertion of thin needles in specific muscles, tendons or fascia, most commonly used to treat pain)	31
Other	9

Commonly, more than one treatment modality was used at each treatment

^a^https://www.gonstead.com.au/for-patients/what-is-gonstead/

^b^https://sommerschiropractic.com/our-chiropractic-technique/

^c^https://en.wikipedia.org/wiki/Activator_technique

^d^https://en.wikipedia.org/wiki/Traction_(orthopedics) (see mechanical traction)

^e^https://mercklingdc.com/2019/10/01/pelvic-blocking-techniques/

^f^https://en.wikipedia.org/wiki/Joint_mobilization

### Responder/non-responder

Demographics of the responder (≥ 2/10 NRS-11) and non-responder (≤ 1/10 NRS-11) groups can be found in Table 1.

Significant improvement in pain intensity was observed between week 2 and 4 (1.1 (0.5–0.8)) for individuals in the non-responder group who received further treatment in the individualized care period, however not considered clinically significant (considered to be 2/10 points in NRS-11).

Among individuals of the responder group who received additional treatment in the individualized care period, statistically significant worsening, or borderline significant worsening in NRS-11 in the whole individualized care period was reported.

For the responder group, the mean change in NRS-11 at the end of the intervention period was 2.7 (1.4) points, which changed to 3.3 (1.8) points after the individually tailored treatment period, showing no clinically significant change post-intervention. Thus, two months after the intervention period ended, the individuals in the responder group who received further treatment in the individualized care period still reported clinically significant improvements from baseline (5.7 (1.4)). Parameter for difference by sex was statistically significant but did not change the conclusions.

Online Appendix 3 contains the changes in NRS-11 for individuals in the responder and non-responder groups who received further treatment in the individualized care period. The table describes changes in the two groups relative to the end of the intervention period (week 2).

Adjusting for age and baseline values did not significantly affect the estimates.

**Fig. 1:**
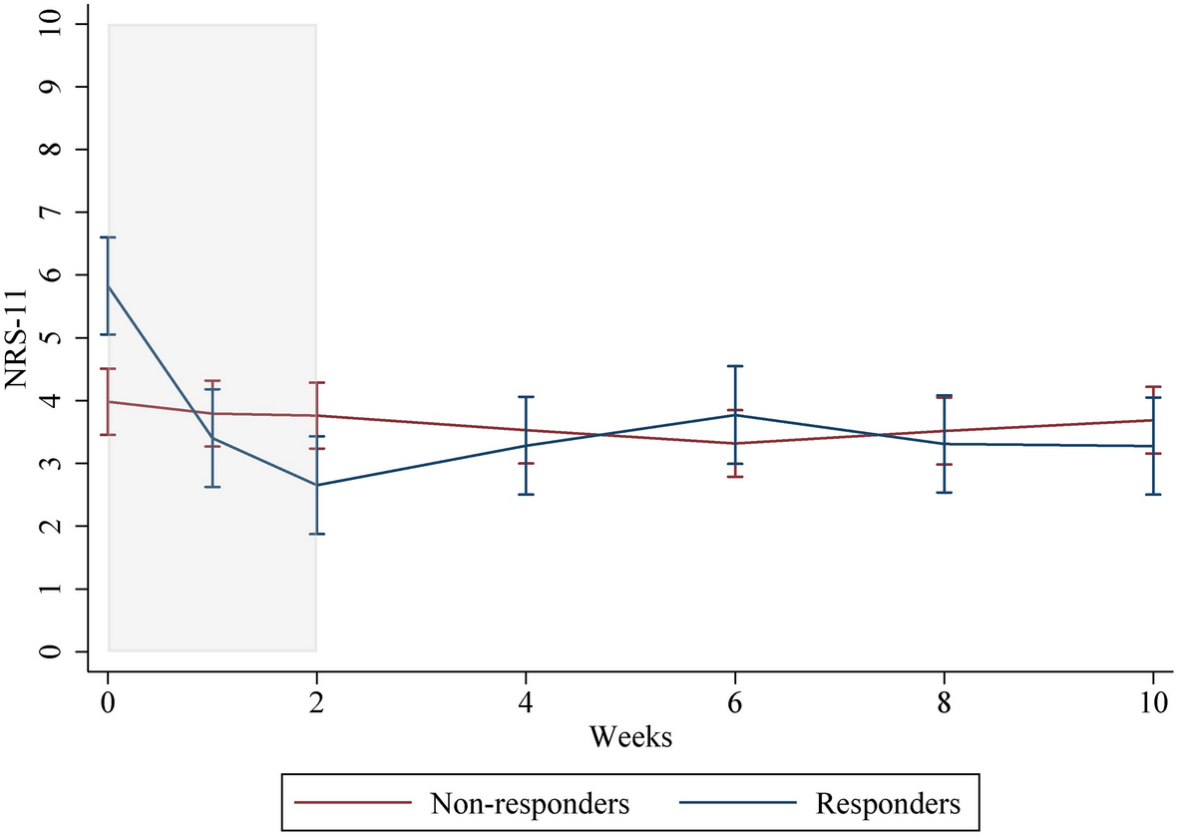
Illustration of mean NRS-11 for each time point during a 2-week intervention and an 8-week individualized care period for responders and non-responders after the intervention. The intervention period is marked in grey

The pain intensity in the individualized care period of the two groups is illustrated in Fig. 1.

Changes in the quality and affective component of pain (McGill questionnaire), neck disability (Neck disability index), and generic health status (EQ-5D) for the individuals in the responder and non-responder groups who received further treatment in the individualized care period are found in Online Appendix 4–6. Statistically significant improvement in the whole individualized care period was found for neck disability among individuals in the non-responder group who received further treatment in the individualized care period. Adjusting for age and BL values did not affect the estimates. A slight statistically significant worsening in quality of life was observed in the first two weeks of the individualized care period among non-responders, and a slight improvement in affected quality of pain in the same two weeks among individuals in the non-responder group who received further treatment in the individualized care period. None of the observed changes were clinically important.

A statistically significant association between the increasing number of treatments and worsening in NRS-11 following the intervention period was observed for individuals in the responder group who received further treatment in the individualized care period, as seen in Table 4. The value of 0.4 refers to the increase in NRS-11 for each added treatment during the individualized care period.

**Table 4:** Association between the number of treatments and change in NRS-11 for individuals in the responder and non-responder groups who received further treatment in the individualized care period

	Responder	*P*-value	CI	
NRS-11 (mean) 10 weeks post-intervention	2.2	0.00	1.4	3.1
Change in NRS-11 (mean) for each applied treatment	0.4	0.03	0.0	0.7

	Non-responder	*P*-value	CI	
NRS-11 (mean) 10 weeks post-intervention	3.4	0.00	2.5	4.4
Change in NRS-11 (mean) for each applied treatment	0.1	0.63	− 0.2	0.4

Adjusting for age, sex, and baseline values affected the estimates for the responder group by reducing the weekly change by half (from 0.4 to 0.2 points). The baseline values significantly contributed to this reduction. Please see Online Appendix 7 for the results from the adjusted model. 

### Intervention/control (intervention period)

No statistically significant difference between the individuals who received further treatment in the pain intensity, disability, affected quality of pain, and quality of life was observed after the 2-month individualized care period for the intervention (SMT and home stretching exercises) and control groups (home stretching exercises only) from the intervention period. The variable gender contributed to the statistical model for NRS-11, NDI, McGill, and EQ-5D but did not change the estimates to any greater degree, as seen in Online Appendix 8–11.

**Fig. 2:**
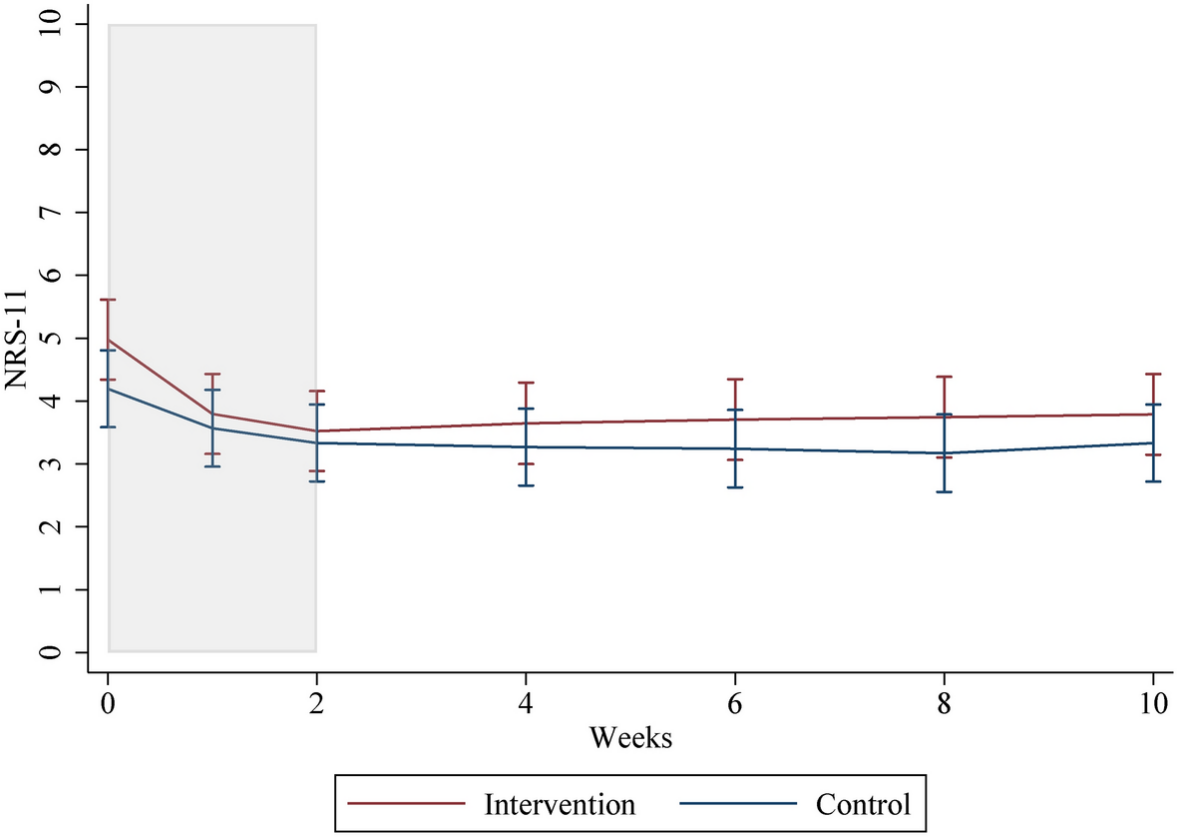
Graph of mean NRS-11 for each time point for intervention and control groups. The intervention period is marked in grey

It was observed that pain intensity remained stable for individuals who received further treatment in both intervention and control groups for two months following the intervention period. This is illustrated in Fig. 2.

Individuals who received further treatment in the intervention group had a mean of 3.1 points (SD: 1.5) treatments during the individualized care period, while among individuals who received further treatment in the control group, a mean of 2.6 points (SD: 1.5) was reported. No statistically significant association was observed between the number of treatments and self-reported pain (NRS-11). Adjusting for age, sex, and BL values did not significantly affect the estimates.

### Females/males

A gender stratification was performed among individuals who received further treatment in the individualized care period as a statistically significant association for sex was observed when investigating intervention/control. Baseline demographics stratified on sex can be found in Table 1.

Females reported more intense NP at baseline (4.8/10 points (95% CI: 4.2, 5.3)) compared to men (4.0/10 points (95% CI: 3.9, 4.5).

**Fig. 3:**
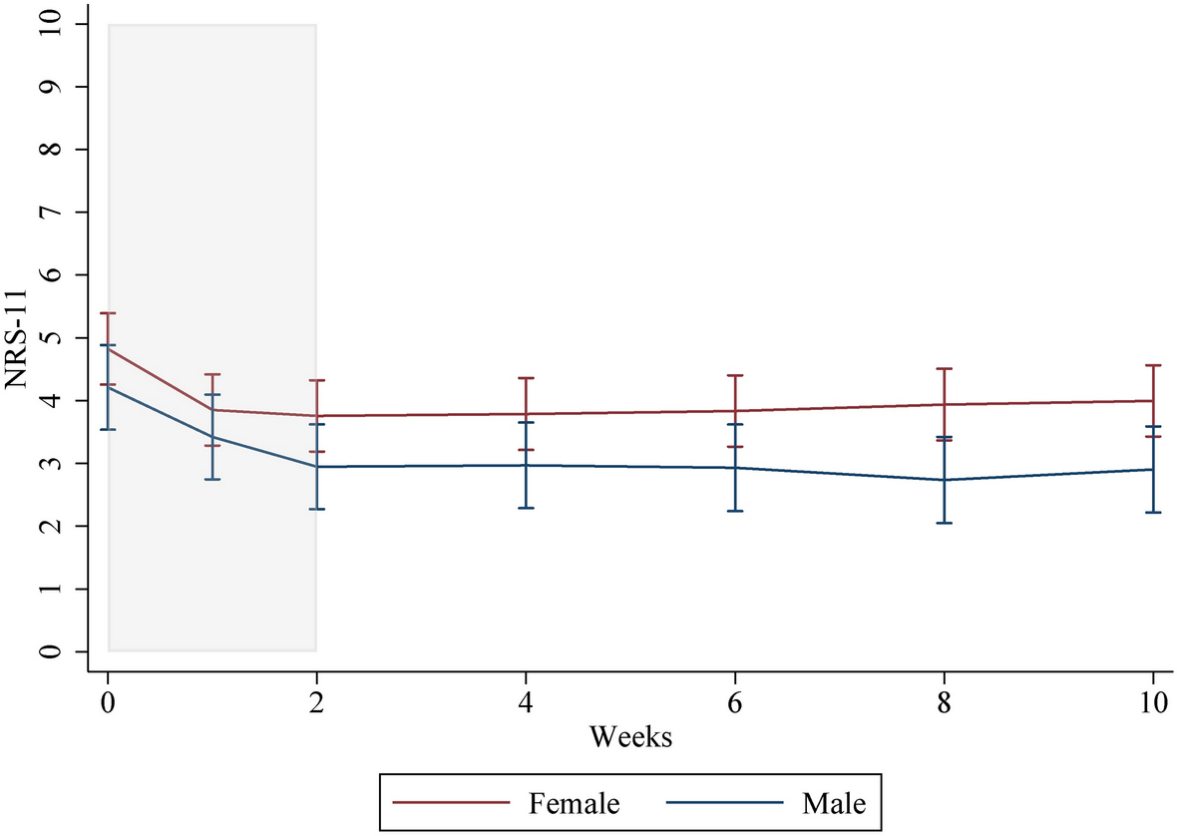
Graph of mean NRS-11 for each time point during the 2-week intervention and the 8-week individualized care period for individuals with NP receiving further treatment, stratified by sex. The intervention period is marked in grey

The improvement in pain intensity among females was 1.1 points (95% SD: 0.5, 1.5) compared to 1.2 points (95% SD: 0.8, 1.6) among males within the first two weeks of interventions and remained stable for the following two months among individuals receiving further treatment. This is illustrated in Fig. 3. No statistically significant changes were observed for either males or females receiving further treatment from the end of the intervention and over the individualized care period.

## Discussion

The study aimed to investigate whether participants improved when receiving individually tailored care after a standardized treatment protocol. Among those receiving additional individually tailored treatments, only significant improvement in pain intensity was found from week 2 to week 4 in the non-responder group, but not within what is assumed to be clinically important. Neck disability, however, significantly improved in the whole individualized care period among the non-responders. Among individuals in the responder group receiving further treatment, significantly increased pain intensity following the intervention period was reported. There was also a significant increase in pain for each added individually tailored treatment, which was not the case after adjusting for age, sex, and baseline values.

A slight improvement in the quality and affective component of pain in the first two weeks of the individualized care period was found among individuals in the non-responder group who received further treatment. The opposite was observed for quality of life.

We expected participants who did not significantly improve in the intervention period to improve in pain intensity when receiving individually tailored treatments. This was only the case from weeks 2 to 4 in the individualized care period. A possible explanation is that the participants who did not improve from the interventions (home stretching exercises with or without SMT) were unable to respond and will not improve, regardless of the intervention. Neck disability did improve in the individualized care period, indicating that individually tailored treatment does influence the disabling consequences of neck pain.

For individuals who received further treatment in the responder group, however, a statistically significant worsening in pain intensity was observed in the individualized care period. Even so, the pain level did not return to baseline levels. The deterioration in pain was assumed to be due to be reflecting normal fluctuations in pain, implying that patients experiencing improvement in the first two weeks will (either by normal fluctuations or due to the interventions) have fluctuating pain with the greatest possibility of worsening again. However, it cannot be ruled out that the continued intervention was responsible for the worsening. Also, this group is rather small [29], causing the mean to be strongly influenced by extreme cases.

An association between an increasing number of treatments and a worsening in pain intensity was found among individuals who received further treatment in the responder group. Considering the findings discussed above, this relationship was expected, mirroring the need for care in this group. Thus, participants with an initial clinically significant improvement would seek care again when experiencing worsening to try to reduce their pain levels.

Previous research has shown that patients tend to improve within the first four treatments and that this improvement is stable over time [17]. Therefore, it was expected that the improvement observed in the intervention period would be stable. For the control group not receiving manual therapy, an improvement from a combination of treatments was expected based on previous research and guidelines suggesting a combination of treatments as the first choice of care [18–21]. Most of the individuals who received individually tailored treatment after the intervention received manual therapy. However, this did not result in such an outcome. It is possible that the contextual factors of seeing the chiropractor, including examinations and conversations, had already resulted in a maximum effect, and including manual therapy was not going to add to a better outcome.

Even though sex had a statistically significant effect on the statistical models for intervention and control, it did not change the conclusion or greatly alter the estimates when adjusted for. Also, stable measurements were observed for both males and females who received further treatment after the intervention period ended, and no association between pain intensity and adding individually tailored treatments was found for females or males.

### Weaknesses

The clinicians performing the interventions could not be blinded to the group allocation. This could have favored the intervention group, but the risk was considered low, considering that the therapists received written and verbal instructions on how to communicate and treat the study participants. The participants were no longer blinded after the intervention period ended.

Many of the participants had been to the clinic before the clinical trial commenced. Most had also experienced a positive effect from chiropractic treatment in the past, likely experiencing a positive effect as they returned for care in this study. This could have led to selection bias, though of an unknown degree.

We did not record whether the patients kept doing the stretching exercises in the individualized care period.

Recruitment to the trial was done up to five weeks before the start of the study, and a relatively large proportion of the participants had low-intensity NP at baseline. Considering that persistent or recurrent NP is a fluctuation condition, the motive for signing up for a study could be a flare-up of symptoms, which would then regress toward the mean before the baseline visit. The rationale behind not setting a minimal pain level is that by excluding participants with low levels of pain, the study group would not represent the diverse group of people with persistent or recurrent neck pain. This could, however, have led to a floor effect.

### Strengths

This was a randomized, well-controlled trial with an excellent response rate (Online Appendix 2). The study population was blinded to what intervention the opposite group was receiving in the intervention period. The research assistant and statistician were blinded to the group allocation.

Emphasis was placed on giving both groups the same time and attention, including “hands-on” palpation for the control group, assuring minimal differences in contextual factors.

### External validity

This study was pragmatic, performed in a clinical setting with treatment options similar to standard treatment strategies, and with individually tailored treatment decided by the clinician in collaboration with the patient, following the intervention period. The study group included individuals with all levels of NP to encompass the diverse group of persistent or recurrent NP patients. Thus, the external validity is deemed to be good.

## Conclusion

Participant who did not respond after a two-week intervention period in a randomized trial of manual therapy and stretching did not improve in pain intensity with individualized care the following two months but did significantly improve in neck disability, implying that individualized care has a greater effect on disability than self-reported pain. The participants who experienced a minimal clinically important improvement in pain intensity from the intervention period slightly worsened over the following eight weeks in pain intensity when receiving further treatments, possibly reflecting regression to mean. For the responders in the trial, worsening in pain was associated with an increasing number of individually tailored treatments following the intervention period, possibly mirroring the need for care.

## Supplementary Information

Supplementary Material 1

## Data Availability

The data supporting this study's findings were used under license for the current study. Restrictions apply to the availability of these data; hence they are not publicly available. Data can, however, be obtained from the authors upon reasonable request to the main author (Anders Galaasen Bakken), and with permission of Karolinska Institutet.

## Abbreviations

NP: Neck pain
SMT: Spinal manipulative therapy
NDI: Neck disability index

## References

[1] Corp N, Mansell G, Stynes S, Wynne-Jones G, Morsø L, Hill JC, et al. Evidence-based treatment recommendations for neck and low back pain across Europe: a systematic review of guidelines. Eur J Pain. 2021;25(2):275–95. 33064878 10.1002/ejp.1679 PMC7839780

[2] Miller J, Gross A, D’Sylva J, Burnie SJ, Goldsmith CH, Graham N, et al. Manual therapy and exercise for neck pain: a systematic review. Man Ther. 2010;15(4):334–54. 20593537

[3] D’Sylva J, Miller J, Gross A, Burnie SJ, Goldsmith CH, Graham N, et al. Manual therapy with or without physical medicine modalities for neck pain: a systematic review. Man Ther. 2010;15(5):415–33. 20538501 10.1016/j.math.2010.04.003

[4] Fredin K, Loras H. Manual therapy, exercise therapy or combined treatment in the management of adult neck pain—a systematic review and meta-analysis. Musculoskelet Sci Pract. 2017;31:62–71. 28750310 10.1016/j.msksp.2017.07.005

[5] Hidalgo B, Hall T, Bossert J, Dugeny A, Cagnie B, Pitance L. The efficacy of manual therapy and exercise for treating non-specific neck pain: a systematic review. J Back Musculoskelet Rehabil. 2017;30(6):1149–69. 28826164 10.3233/BMR-169615 PMC5814665

[6] Coulter ID, Crawford C, Vernon H, Hurwitz EL, Khorsan R, Booth MS, et al. Manipulation and mobilization for treating chronic nonspecific neck pain: a systematic review and meta-analysis for an appropriateness panel. Pain Phys. 2019;22(2):E55–70. PMC6800035 30921975

[7] Vernon H, Humphreys K, Hagino C. Chronic mechanical neck pain in adults treated by manual therapy: a systematic review of change scores in randomized clinical trials. J Manip Physiol Ther. 2007;30(3):215–27. 10.1016/j.jmpt.2007.01.014 17416276

[8] Leaver AM, Refshauge KM, Maher CG, McAuley JH. Conservative interventions provide short-term relief for non-specific neck pain: a systematic review. J Physiother. 2010;56(2):73–85. 20482474 10.1016/s1836-9553(10)70037-0

[9] Vincent K, Maigne J-Y, Fischhoff C, Lanlo O, Dagenais S. Systematic review of manual therapies for nonspecific neck pain. Joint Bone Spine. 2013;80(5):508–15. 23165183 10.1016/j.jbspin.2012.10.006

[10] Galaasen Bakken A, Axén I, Eklund A, O’Neill S. The effect of spinal manipulative therapy on heart rate variability and pain in patients with chronic neck pain: a randomized controlled trial. Trials. 2019;20(1):590. 31606042 10.1186/s13063-019-3678-8 PMC6790043

[11] Galaasen Bakken A, Eklund A, Hallman DM, Axén I. The effect of spinal manipulative therapy and home stretching exercises on heart rate variability in patients with persistent or recurrent neck pain: a randomized controlled trial. Chiropr Man Ther. 2021;29(1):48. 10.1186/s12998-021-00406-0 PMC8628060 34844625

[12] Bakken AG, Eklund A, Warnqvist A, O’Neill S, Axen I. The effect of two weeks of spinal manipulative therapy and home stretching exercises on pain and disability in patients with persistent or recurrent neck pain; a randomized controlled trial. BMC Musculoskelet Disord. 2021;22(1):903. 34706706 10.1186/s12891-021-04772-x PMC8549416

[13] Bakken AG, Eklund A, Warnqvist A, O’Neill S, Hallman DM, Axén I. Are changes in pain associated with changes in heart rate variability in patients treated for recurrent or persistent neck pain? BMC Musculoskelet Disord. 2022;23(1):895. 36192738 10.1186/s12891-022-05842-4 PMC9531383

[14] Schellingerhout JM, Verhagen AP, Heymans MW, Pool JJM, Vonk F, Koes BW, et al. Which subgroups of patients with non-specific neck pain are more likely to benefit from spinal manipulation therapy, physiotherapy, or usual care? Pain. 2008;139(3):670–80. 18774225 10.1016/j.pain.2008.07.015

[15] Henchoz Y, Kai-Lik SA. Exercise and nonspecific low back pain: a literature review. Joint Bone Spine. 2008;75(5):533–9. 18801686 10.1016/j.jbspin.2008.03.003

[16] Dueñas L, Aguilar-Rodríguez M, Voogt L, Lluch E, Struyf F, Mertens MGCAM, et al. Specific versus non-specific exercises for chronic neck or shoulder pain: a systematic review. J Clin Med. 2021;10(24):5946. 34945241 10.3390/jcm10245946 PMC8706212

[17] Leboeuf-Yde C, Grønstvedt A, Borge JA, Lothe J, Magnesen E, Nilsson Ø, et al. The nordic back pain subpopulation program: demographic and clinical predictors for outcome in patients receiving chiropractic treatment for persistent low back pain. J Manip Physiol Ther. 2004;27(8):493–502. 10.1016/j.jmpt.2004.08.001 15510092

[18] Bussieres AE, Stewart G, Al-Zoubi F, Decina P, Descarreaux M, Hayden J, et al. The treatment of neck pain-associated disorders and whiplash-associated disorders: a clinical practice guideline. J Manip Physiol Ther. 2016;39(8):523-64.e27. 10.1016/j.jmpt.2016.08.007 27836071

[19] Bryans R, Decina P, Descarreaux M, Duranleau M, Marcoux H, Potter B, et al. Evidence-based guidelines for the chiropractic treatment of adults with neck pain. J Manip Physiol Ther. 2014;37(1):42–63. 10.1016/j.jmpt.2013.08.010 24262386

[20] Blanpied PR, Gross AR, Elliott JM, Devaney LL, Clewley D, Walton DM, et al. Neck pain: revision 2017. J Orthop Sports Phys Ther. 2017;47(7):A1-a83. 28666405 10.2519/jospt.2017.0302

[21] Parikh P, Santaguida P, Macdermid J, Gross A, Eshtiaghi A. Comparison of CPG’s for the diagnosis, prognosis and management of non-specific neck pain: a systematic review. BMC Musculoskelet Disord. 2019;20(1):81. 30764789 10.1186/s12891-019-2441-3 PMC6376764

[22] Hutting N, Kerry R, Coppieters MW, Scholten-Peeters GGM. Considerations to improve the safety of cervical spine manual therapy. Musculoskelet Sci Pract. 2018;33:41–5. 29153924 10.1016/j.msksp.2017.11.003

[23] Williamson A, Hoggart B. Pain: a review of three commonly used pain rating scales. J Clin Nurs. 2005;14(7):798–804. 16000093 10.1111/j.1365-2702.2005.01121.x

[24] Jensen MP, Karoly P, Braver S. The measurement of clinical pain intensity: a comparison of six methods. Pain. 1986;27(1):117–26. 3785962 10.1016/0304-3959(86)90228-9

[25] Dworkin RH, Turk DC, Trudeau JJ, Benson C, Biondi DM, Katz NP, et al. Validation of the short-form McGill pain questionnaire-2 (SF-MPQ-2) in acute low back pain. J Pain. 2015;16(4):357–66. 25640290 10.1016/j.jpain.2015.01.012

[26] Burckhardt CS, Bjelle A. A Swedish version of the short-form McGill Pain Questionnaire. Scand J Rheumatol. 1994;23(2):77–81. 8165442 10.3109/03009749409103032

[27] Dansie EJ, Turk DC. Assessment of patients with chronic pain. Br J Anaesth. 2013;111(1):19–25. 23794641 10.1093/bja/aet124 PMC3841375

[28] Bjork S, Norinder A. The weighting exercise for the Swedish version of the EuroQol. Health Econ. 1999;8(2):117–26. 10342725 10.1002/(sici)1099-1050(199903)8:2<117::aid-hec402>3.0.co;2-a

[29] Brooks R. EuroQol: the current state of play. Health policy (Amsterdam, Netherlands). 1996;37(1):53–72. 10158943 10.1016/0168-8510(96)00822-6

[30] Ackelman BH, Lindgren U. Validity and reliability of a modified version of the neck disability index. J Rehabil Med. 2002;34(6):284–7. 12440803 10.1080/165019702760390383

[31] Vartiainen P, Mäntyselkä P, Heiskanen T, Hagelberg N, Mustola S, Forssell H, et al. Validation of EQ-5D and 15D in the assessment of health-related quality of life in chronic pain. Pain. 2017;158(8):1577–85. 28715354 10.1097/j.pain.0000000000000954

[32] Van Buuren S, Brand JPL, Groothuis-Oudshoorn CGM, Rubin DB. Fully conditional specification in multivariate imputation. J Stat Comput Simul. 2006;76(12):1049–64.

[33] Farrar JT, Young JP Jr, LaMoreaux L, Werth JL, Poole RM. Clinical importance of changes in chronic pain intensity measured on an 11-point numerical pain rating scale. Pain. 2001;94(2):149–58. 11690728 10.1016/S0304-3959(01)00349-9

[34] Hawker GA, Mian S, Kendzerska T, French M. Measures of adult pain: Visual Analog Scale for Pain (VAS Pain), Numeric Rating Scale for Pain (NRS Pain), McGill Pain Questionnaire (MPQ), Short-Form McGill Pain Questionnaire (SF-MPQ), Chronic Pain Grade Scale (CPGS), Short Form-36 Bodily Pain Scale (SF-36 BPS), and Measure of Intermittent and Constant Osteoarthritis Pain (ICOAP). Arthritis Care Res. 2011;63(Suppl 11):S240–52. 10.1002/acr.20543 22588748

[35] https://etikprovningsmyndigheten.se/ [Available from: https://etikprovningsmyndigheten.se/.

[36] Charlton E. Ethical guidelines for pain research in humans. Committee on Ethical Issues of the International Association for the Study of Pain. Pain. 1995;63(3):277–8. 8719527 10.1016/0304-3959(95)90040-3

