# Supplementary material for: The response to individualized treatment after a standardized treatment protocol among neck pain sufferers: a secondary analysis of a randomized controlled trial
Source: Chiropr Man Therap. 2025 Apr 11;33:13. doi: 10.1186/s12998-025-00579-y (PMC11987369; doi:10.1186/s12998-025-00579-y)
Supplement: Supplementary file 1 — Supplementary Material 1 [file 12998_2025_579_MOESM1_ESM.docx]

**Appendix 1. Questionnaire sent by Email**

6.4: Nacksmärtans **intensitet** (i genomsnitt)det senaste dygnet

Inte alls ont Outhärdligt ont 0 1 2 3 4 5 6 7 8 9 10

🞎 🞎 🞎 🞎 🞎 🞎 🞎 🞎 🞎 🞎 🞎

| 6.5: **Beskriv** smärtan i nacken som du känner just nu: (sätt kryss) | Ingen | Lindrig | Måttlig | Uttalad |
| --- | --- | --- | --- | --- |
| Pulserande |  |  |  |  |
| Blixtrande |  |  |  |  |
| Stickande |  |  |  |  |
| Skärande |  |  |  |  |
| Krampaktig |  |  |  |  |
| Gnagande |  |  |  |  |
| Brännande |  |  |  |  |
| Molande |  |  |  |  |
| Tung |  |  |  |  |
| Ömmande |  |  |  |  |
| Sprängande |  |  |  |  |
| Utmattande |  |  |  |  |
| Kväljande |  |  |  |  |
| Fasansfull |  |  |  |  |
| Straffande-grym |  |  |  |  |

6.10: Nuvarande smärtintensitet (ringa in ditt svar):

0 – Ingen smärta

1 – Lindrig

2 – Obehaglig

3 – Besvärlig

4 – Fruktansvärd

5 - Outhärdlig

Markera, genom att kryssa i en ruta i varje nedanstående grupp, vilket påstående som bäst beskriver Ditt hälsotillstånd **i dag**.

**11.1: RÖRELSE**

🞏 Jag går utan svårigheter

🞏 Jag kan gå men med viss svårighet

🞏 Jag är sängliggande

**11.2: PERSONLIG OMVÅRDNAD**

🞏 Jag behöver ingen hjälp med min dagliga hygien, mat eller påklädning

🞏 Jag har vissa problem att tvätta eller klä mig själv

🞏 Jag kan inte tvätta eller klä mig själv

**11.3: DAGLIGA AKTIVITETER** *(ex arbete, studier, hushållssysslor, familj eller fritid)*

🞏 Jag klarar av min huvudsakliga sysselsättning

🞏 Jag har vissa problem med att klara av min huvudsakliga sysselsättning

🞏 Jag klarar inte av min huvudsakliga sysselsättning

**11.4: SMÄRTA / BESVÄR**

🞏 Jag har varken smärtor eller besvär

🞏 Jag har måttliga smärtor eller besvär

🞏 Jag har svåra smärtor eller besvär


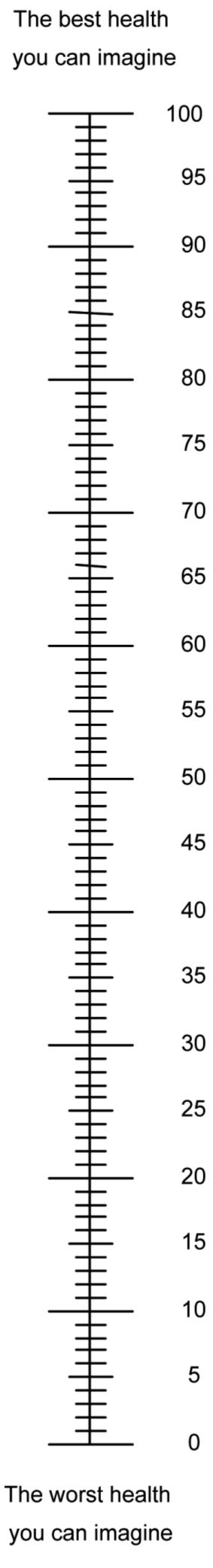
**11.5: ÅNGEST / DEPRESSION**

🞏 Jag är inte orolig eller nedstämd

🞏 Jag är orolig eller nedstämd i viss utsträckning

🞏 Jag är i högsta grad orolig eller nedstämd

**11.6: Hur tycker du att Din hälsa är I DAG?**

**S**kalan till höger går från 0 till 100.

‐ 100 är den bästa hälsa du kan tänka dig.

‐ 0 är den sämsta hälsa du kan tänka dig.

‐ Markera med X på skalan för att indikera hur din hälsa är I DAG.

Var god att också skriv motsvarande siffra här:

DIN HÄLSA I DAG= _____________

**NACKFUNKTIONSSKALA**

Följande frågor är utformade för att ge oss information om hur din nacksmärta påverkar ditt dagliga liv. Besvara varje avsnitt och markera bara den enda ruta som passar dig. Vi är medvetna om att det kan vara svårt att välja mellan två närstående påståenden, men var vänlig kryssa bara i den rutan som mest motsvarar er situation.

12.1: SMÄRTINTENSITET

🞏 Jag har ingen smärta för närvarande

🞏 Smärtan är mycket lätt

🞏 Smärtan är måttlig

🞏 Smärtan är svår

🞏 Smärtan är mycket svår

🞏 Smärtan är värsta tänkbara

12.2: PERSONLIG OMVÅRDNAD (Hygien, påklädning etc)

🞏 Jag kan sköta mig själv som vanligt utan att få ökad smärta

🞏 Jag kan sköta mig själv som vanligt, men det orsakar ökad smärta

🞏 Det innebär smärta att sköta mig själv och jag är försiktig och långsam

🞏Jag behöver en del hjälp, men klarar det mesta av min personliga omvårdnad

🞏 Jag behöver hjälp varje dag med det mesta i min personliga omvårdnad

🞏 Jag klär inte på mig, tvättar mig med svårigheter och ligger till sängs

12.3: LYFTA

🞏 Jag kan lyfta tunga saker utan ökad smärta

🞏 Jag kan lyfta tunga saker, men det ger ökad smärta

🞏 Smärtan hindrar mig från att lyfta tunga föremål från golvet, men jag klarar det om det är lämpligt placerat, ex på ett bord

🞏 Smärtan hindrar mig från att lyfta tunga föremål, men jag klarar medeltunga föremål, om

de är lämpligt placerade

🞏 Jag kan lyfta mycket lätta föremål

🞏 Jag kan inte lyfta eller bära något överhuvudtaget

12.4: LÄSNING

🞏 Jag kan läsa så mycket som jag vill utan smärta från nacken

🞏 Jag kan läsa så mycket jag vill med lätt smärta i nacken

🞏 Jag kan läsa så mycket jag vill, men med måttlig smärta i nacken

🞏 Jag kan inte läsa så mycket jag vill p g a måttlig smärta från nacken

🞏 Jag kan knappast läsa alls p g a svår smärta från nacken

🞏 Jag kan inte läsa alls p g a smärtan

12.5: HUVUDVÄRK

🞏 Jag har ingen huvudvärk överhuvudtaget

🞏 Jag har lätt huvudvärk då och då

🞏 Jag har måttlig huvudvärk då och då

🞏 Jag har måttlig huvudvärk ofta

🞏 Jag har svår huvudvärk ofta

🞏 Jag har svår huvudvärk praktiskt taget hela tiden

12.6: KONCENTRATION

🞏 Jag kan koncentrera mig helt och hållet när jag behöver, utan problem

🞏 Jag kan koncentrera mig helt och hållet när jag behöver, men får lindriga besvär

🞏 Jag har måttliga svårigheter att koncentrera mig när jag behöver

🞏 Jag har stora svårigheter att koncentrera mig när jag behöver

🞏 Jag har avsevärda problem att koncentrera mig när jag behöver

🞏 Jag kan inte koncentrera mig alls

12.7: ARBETE

🞏 Jag kan utföra så mycket arbete som jag vill

🞏 Jag kan bara göra mitt vanliga arbete, men inte mer

🞏 Jag kan göra det mesta av mitt vanliga arbete, men inte mer

🞏 Jag kan inte utföra mitt vanliga arbete

🞏 Jag kan knappast utföra något arbete alls

🞏 Jag kan inte utföra något arbete alls

12.8: BILKÖRNING

🞏 Jag kan köra bil utan någon nacksmärta

🞏 Jag kan köra bil så länge jag vill, med lätt smärta i nacken

🞏 Jag kan köra bil så länge jag vill, med måttlig smärta i nacken

🞏 Jag kan inte köra bil så länge jag vill p g a måttlig smärta från nacken

🞏 Jag kan knappast köra bil alls p g a svår smärta från nacken

🞏 Jag kan inte köra bil alls p g a nacksmärtan

12.9: SÖMN

🞏 Jag har inga problem med sömnen

🞏 Min sömn är lätt störd (mindre än 1 timme sömnlöshet pga. smärtan)

🞏 Min sömn är måttligt störd (1-2 timmer sömnlöshet pga. smärtan)

🞏 Min sömn är tämligen störd (2-3 timmer sömnlöshet pga. smärtan)

🞏 Min sömn är kraftigt störd (3-5 timmer sömnlöshet pga. smärtan)

🞏 Min sömn är helt och hållet störd (5-7 timmer sömnlöshet pga. smärtan)

12.10: FRITIDSAKTIVITETER

🞏Jag klarar att utföra alla mina fritidsaktiviteter utan någon nacksmärta

🞏 Jag klarar att utföra alla mina fritidsaktiviteter, men med lätt smärta i nacken

🞏 Jag klarar att utföra de flesta, dock inte alla mina vanliga fritidsaktiviteter pga. smärta i nacken

🞏 Jag klarar bara att utföra ett fåtal av mina vanliga fritidsaktiviteter pga. smärta i nacken

🞏 Jag kan knappast utföra några fritidsaktiviteter pga. smärta i nacken

🞏 Jag kan inte utföra några fritidsaktiviteter alls

Tänk på de 2 senaste veckorna när du svarar på följande frågor:

|  |  | **Instämmer inte** | **Instämmer** |
| --- | --- | --- | --- |
|  |  | 0 | 1 |
| 13.1: | Min nacksmärta har **strålat ut i min arm/mina armar** vid något tillfälle de senaste 2 veckorna. | □ | □ |
| 13.2: | Jag har haft smärta i **ländryggen** vid något tillfälle de senaste 2 veckorna | □ | □ |
| 13.3: | Jag har bara **gått korta sträckor** på grund av min nacksmärta. | □ | □ |
| 13.4: | Under de senaste 2 veckorna har det tagit **längre tid än vanligt att klä mig** på grund av nacksmärtan. | □ | □ |
| 13.5: | Det kan vara skadligt för en person med mina besvär att vara fysiskt aktiv | □ | □ |
| 13.6: | Jag har haft oroande tankar en stor del av tiden. | □ | □ |
| 13.7: | Jag upplever att **min nacksmärta är fruktansvärd** och att den **aldrig kommer att bli bättre.** | □ | □ |
| 13.8: | I allmänhet har jag inte **glatt mig över** de saker som jag brukar glädja mig åt. | □ | □ |

13.9: På det stora hela, hur **besvärlig** har din nacksmärta varit **de senaste 2 veckorna**?

| Inte alls | Lätt | Måttligt | Väldigt mycket | Extremt |
| --- | --- | --- | --- | --- |
| □ | □ | □ | □ | □ |

Appendix 2. The proportion of the number of questions answered at each time point following the intervention period.

|  | 2 weeks | 4 weeks | 6 weeks | 8 weeks | Total |
| --- | --- | --- | --- | --- | --- |
| NRS-11 | 119/127 | 120/127 | 118/127 | 122/127 | 479/508 |
| % | 94% | 94% | 93% | 96% | 94% |
| McGill | 1522/1905 | 1574/1905 | 1599/1905 | 1668/1905 | 6363/7620 |
| % | 80% | 83% | 84% | 88% | 84% |
| NDI | 1176/1270 | 1220/1270 | 1180/1270 | 1230/1270 | 4806/5080 |
| % | 93% | 96% | 93% | 97% | 95% |
| EQ-5D | 587/635 | 613/635 | 583/635 | 612/635 | 2395/2540 |
| % | 92% | 97% | 92% | 96% | 94% |
| Total | 3404/3973 | 3527/3973 | 3480/3973 | 3632/3973 | 14043/15748 |
| % | 86% | 89% | 88% | 91% | 89% |

Appendix 3. Changes in NRS-11 for the individuals receiving additional treatments in the responder and non-responder groups in the follow-up period with the end of the intervention period (week two) as reference.

| Responders | B | CI |  | P-value |
| --- | --- | --- | --- | --- |
| Week 4 | 0.6 | -0.0 | 1.3 | 0.05 |
| Week 6 | 1.1 | 0.5 | 1.8 | 0.00 |
| Week 8 | 0.7 | 0.0 | 1.3 | 0.04 |
| Week 10 | 0.6 | -0.0 | 1.3 | 0.05 |
| Non-responders | B | CI |  | P-value |
| We-ek 4 | -0.2 | -0.7 | 0.2 | 0.28 |
| Week 6 | -0.5 | -0.9 | -0.0 | 0.03 |
| Week 8 | -0.2 | -0.6 | 0.2 | 0.26 |
| Week 10 | -0.05 | -0.5 | 0.4 | 0.80 |

Appendix 4. B-coefficient for McGill Questionnaire among responders and non-responders receiving additional treatments in the in the follow-up period.

| Responders | B | CI | | P-value |
| --- | --- | --- | --- | --- |
| Week 4 | 1.5 | -0.9 | 3.8 | 0.21 |
| Week 6 | 2.3 | -0.0 | 4.6 | 0.05 |
| Week 8 | 1.8 | -0.5 | 4.0 | 0.13 |
| Week 10 | 0.7 | -1.6 | 3.0 | 0.6 |
| Non-responders | B | CI | | P-value |
| Week 4 | -1.4 | -2.9 | -0.0 | 0.05 |
| Week 6 | -1.4 | -2.8 | 0.1 | 0.06 |
| Week 8 | -0.8 | -2.3 | 0.6 | 0.25 |
| Week 10 | 0.3 | -1.2 | 1.7 | 0.72 |

Appendix 5. B-coefficient for Neck Disability Index among responders and non-responders receiving additional treatments in the in the follow-up period.

| Responders | B | CI | | P-value |
| --- | --- | --- | --- | --- |
| Week 4 | 0.4 | -0.8 | 1.5 | 0.54 |
| Week 6 | 0.3 | -0.8 | 1.4 | 0.60 |
| Week 8 | 0.1 | -1.0 | 1.2 | 0.87 |
| Week 10 | -0.4 | -1.6 | 0.7 | 0.43 |
| Non-responders | B | CI | | P-value |
| Week 4 | -1.4 | -2.3 | -0.4 | 0.01 |
| Week 6 | -1.6 | -2.6 | -0.7 | 0.00 |
| Week 8 | -1.2 | -2.2 | -0.3 | 0.01 |
| Week 10 | -1.5 | -2.4 | -0.6 | 0.00 |

Appendix 6. B-coefficient for EQ-5D among responders and non-responders receiving additional treatments in the in the follow-up period.

| Responders | B | CI | | P-value |
| --- | --- | --- | --- | --- |
| Week 4 | -0.01 | -0.03 | 0.01 | 0.27 |
| Week 6 | -0.01 | -0.03 | 0.01 | 0.26 |
| Week 8 | -0.02 | -0.04 | 0.00 | 0.06 |
| Week 10 | -0.00 | -0.02 | 0.02 | 0.77 |
| Non-responders | B | CI | | P-value |
| Week 4 | 0.02 | 0.00 | 0.04 | 0.02 |
| Week 6 | 0.01 | -0.01 | 0.03 | 0.23 |
| Week 8 | 0.01 | -0.01 | 0.02 | 0.59 |
| Week 10 | 0.01 | -0.01 | 0.03 | 0.17 |

Appendix 7. Association between number of treatments and change in NRS-11 for the individuals receiving additional treatments in the responder group in the follow-up period, adjusted for age, sex, and gender.

|  | Responder | P-value | CI | |
| --- | --- | --- | --- | --- |
| NRS-11 after 10 weeks | 1.3 | 0.97 | -62.3 | 64.9 |
| Sex | -0.7 | 0.18 | -1.7 | 0.3 |
| Age | -0.0 | 0.96 | -0.0 | 0.0 |
| Baseline NRS-11 | 0.6 | 0.00 | 0.3 | 0.9 |
| Change in NRS-11 for each applied treatment | 0.2 | 0.14 | -0.1 | 0.5 |

Appendix 8. B-coefficient for NRS-11 for the difference between individuals receiving additional treatments in the intervention/control groups at all time points with the control group as reference.

| BL control group | 4.7 |  | | |
| --- | --- | --- | --- | --- |
| NRS-11 | B-coefficient | CI | | P-value |
| Baseline difference | -0.5 | -1.2 | 0.3 | 0.21 |
| Group/week 1 | 0.09 | -0.5 | 0.7 | 0.78 |
| Group/week 2 | 0.23 | -0.4 | 0.9 | 0.46 |
| Group/week 4 | 0.13 | -0.5 | 0.8 | 0.69 |
| Group/week 6 | -0.14 | -0.8 | 0.5 | 0.67 |
| Group/week 8 | -0.14 | -0.8 | 0.5 | 0.67 |
| Group/week 10 | 0.14 | -0.5 | 0.8 | 0.65 |

Appendix 9. B-coefficient for McGill for the difference between individuals receiving additional treatments in the intervention/control groups at all time points with the control group as reference.

| BL control group | 23.5 |  | | |
| --- | --- | --- | --- | --- |
| McGill | B-coefficient | CI | | P-value |
| Baseline difference | -0.4 | -3.3 | 2.5 | 0.78 |
| Group/week 1 | - 1.0 | -0.3 | 1.3 | 0.38 |
| Group/week 2 | - 1.0 | -0.3 | 1.2 | 0.37 |
| Group/week 4 | -0.1 | -2.4 | 2.2 | 0.94 |
| Group/week 6 | - 1.2 | -3.4 | 1.1 | 0.32 |
| Group/week 8 | -1.4 | -3.7 | 0.9 | 0.24 |
| Group/week 10 | -1.6 | -3.9 | 0.7 | 0.16 |

Appendix 10. B-coefficient for NDI for the difference between individuals receiving additional treatments in the intervention/control groups at all time points with the control group as reference.

| BL control group | 22.9 |  | | |
| --- | --- | --- | --- | --- |
| NDI | B-coefficient | CI | | P-value |
| Baseline difference | -1.0 | -3.5 | 1.5 | 0.42 |
| Group/week 1 | 0.8 | -1.4 | 1.6 | 0.92 |
| Group/week 2 | 0.4 | -1.1 | 1.9 | 0.60 |
| Group/week 4 | 1.1 | -0.34 | 2.7 | 0.14 |
| Group/week 6 | -0.0 | -1.5 | 1.4 | 0.96 |
| Group/week 8 | 0.5 | -1.0 | 2.1 | 0.49 |
| Group/week 10 | 0.8 | -0.7 | 2.3 | 0.31 |

Appendix 11. B-coefficient for EQ-5D for the difference between individuals receiving additional treatments in the intervention/control groups at all time points with the control group as reference.

| BL control group | 0.90 |  | | |
| --- | --- | --- | --- | --- |
| EQ-5D | B-coefficient | CI | | P-value |
| Baseline difference | 0.00 | -0.03 | 0.04 | 0.78 |
| Group/week 1 | 0.00 | -0.02 | 0.02 | 0.87 |
| Group/week 2 | -0.00 | -0.02 | 0.02 | 0.75 |
| Group/week 4 | -0.01 | -0.03 | 0.01 | 0.26 |
| Group/week 6 | -0.01 | -0.03 | 0.01 | 0.46 |
| Group/week 8 | -0.01 | -0.03 | 0.01 | 0.28 |
| Group/week 10 | 0.00 | -0.02 | 0.02 | 0.74 |
